# Therapeutic potential of topical administration of acriflavine against hypoxia-inducible factors for corneal fibrosis

**DOI:** 10.3389/fphar.2022.996635

**Published:** 2022-10-21

**Authors:** Shuyan Zhu, Huimin Shan, Jianqiao Li, Lijie Pan, Shudan Wang, Jing Zhu, Hui Guo, Fenghua Mi, Xinyi Wu, Jia Yin, Kunpeng Pang

**Affiliations:** ^1^ Xi’an People’s Hospital (Xi’an Fourth Hospital), Shanxi Eye Hospital, Xi’an, Shanxi, China; ^2^ Department of Ophthalmology, Qilu Hospital of Shandong University, Jinan, Shandong, China; ^3^ Department of Ophthalmology, Harvard Medical School, Schepens Eye Research Institute of Massachusetts Eye and Ear, Boston, MA, United States

**Keywords:** HIF, corneal fibrosis, corneal hypoxia, TGF-β1, extracellular matrix

## Abstract

Transdifferentiation of keratocytes into fibroblasts or further into myofibroblasts, which produced denser and more disorganized extracellular matrix, is the major cause of corneal fibrosis and scarring, leading to corneal blindness. TGF-β1 is the critical cytokine for the myofibroblast’s transdifferentiation and survival. Hypoxia Inducible Factor (HIF) was found to play an important role in promoting fibrosis in lung, kidney, and dermal tissues recently. Our preliminary study demonstrated that topical administration of the acriflavine (ACF), a drug inhibiting HIF dimerization, delayed corneal opacity and neovascularization after the alkali burn. To know whether ACF could prevent corneal fibrosis and improve corneal transparency, we created a mouse mechanical corneal injury model and found that topical administration of ACF significantly inhibited corneal fibrosis at day 14 post-injury. The reduction of myofibroblast marker α-SMA, and fibronectin, one of the disorganized extracellular matrix molecules, in the corneal stroma were confirmed by the examination of immunohistochemistry and real-time PCR. Furthermore, the ACF inhibited the expression of α-SMA and fibronectin in both TGF-β1 stimulated or unstimulated fibroblasts *in vitro*. This effect was based on the inhibition of HIF signal pathways since the levels of the HIF-1α downstream genes including Slc2a1, Bnip3 and VEGFA were downregulated. To our knowledge, this is the first time to implicate that HIFs might be a new treatment target for controlling corneal fibrosis in mechanical corneal injuries.

## Introduction

The cornea is an avascular and transparent tissue that covers the front of the eye and is responsible for appropriately 70% of the refractive power of the eye. The corneal fibrosis is the third most common cause of blindness, following cataracts and glaucoma (Ibrahim Al-Mashahedah et al., 2019). The risk factors for corneal fibrosis are highly complex and encompass a wide variety of factors such as physical trauma to the eye, chemical burns, infections, refractive surgery, etc. (Karamichos et al., 2010; Wilson et al., 2012; Ibrahim Al-Mashahedah et al., 2019). After the injury, the keratocytes that existed in the corneal stroma are stimulated to proliferate and differentiate into fibroblasts. In some wound sites, the fibroblasts are stimulated by inflammatory factors or cytokines such as transforming growth factor β (particularly TGF-β1 and TGF-β2) and further differentiate into the α -smooth muscle actin (α-SMA) expressed myofibroblasts, which produced denser and more disorganized extracellular matrix (e.g., fibronectin, etc.), resulting in corneal fibrosis and scarring (Gupta et al., 2011; Mittal et al., 2016). At present, treatment options are limited and consist primarily of corneal transplantation.

As an avascular tissue, the cornea is unique in consumption by acquiring oxygen from the atmosphere and is sensitive to the oxygen concentration change (Pang et al., 2021). Hypoxia caused by the contact lens wearing, inflammation, wounds and infections, results in the disruption of corneal epithelial barrier function, delay of the wound healing, alteration of the extracellular matrix, and leads to corneal neovascularization and opacity (Lee et al., 2020; Onochie et al., 2020; Bai et al., 2021). Moreover, the enhancement of hypoxia in the myofibroblast transdifferentiation has been found in many tissues, such as sclera (Wu et al., 2018), skin (Zhao et al., 2017), artery adventitia (Short et al., 2004), and nasal polyps (Moon et al., 2012). The widely known cellular response to oxygen changes is the hypoxia-inducible factors (HIFs). HIF is a heterodimeric complex comprising one of three major oxygen labile HIF-α subunits (HIF-1α, HIF-2α, or HIF-3α) dimerized with HIF-1β to form HIF-1, HIF-2, or HIF-3 transcriptional complexes, respectively (Koh and Powis, 2012). Under hypoxia or inflammatory conditions, HIF-α is stabilized and translocated to the nucleus, where it dimerizes with HIF-1β to transactivate a large number of target genes including those promoting angiogenesis (VEGFA), metabolism (GLUT1/3), cell proliferation and survival (Bnip3, TGF-α), etc. (Corcoran and O’Neil, 2016; Pullamsetti et al., 2020; Yang et al., 2020). Interestingly, multiple studies revealed that HIF played a critical role in promoting fibrosis (Basu et al., 2011; Brereton et al., 2022; Yuan et al., 2022). TGF-β has been reported to cause HIF stabilization (Basu et al., 2011). Noteworthily, the elevated mRNA level of HIFs has also been noted in injured (our preliminary study) or infected corneal tissues (Rao and Suvas, 2019). However, the effects and mechanisms of HIFs in the cornea are unknown and need to be further investigated.

In this study, we found that alkali burn led to hypoxia in the mice’s anterior chamber and promoted the elevation of HIF-1α mRNA levels in the cornea. In order to know how HIFs affected the ocular outcomes after the burn, we treated the injured mice’s eyes with the acriflavine (ACF), a drug inhibiting HIF dimerization by binding to the PAS-B subdomain of HIF-1α and HIF-2α, thereby leading to the inhibition of HIF DNA-binding and transcriptional activity (Lee et al., 2009). We found that ACF treatment delayed the corneal opacity and neovascularization after the injury. However, due to the complex mechanisms of corneal opacity resulting from the alkali-burn-induced limbal stem cell deficiency model, we created a mechanical corneal injury model as described previously to observe how it attenuated the corneal opacity (Mittal et al., 2016). We found that topical administration of ACF restored the corneal transparency during 28 days of follow-up, and inhibited the generation of fibrosis in the injured corneal stroma. Furthermore, ACF reduced the mRNA level of α-SMA and fibronectin in the corneal fibroblasts stimulated by TGF-β1, and downregulated the levels of HIF-1α downstream genes. To our knowledge, this was the first time to demonstrate that HIF signaling pathways played an important role in corneal fibrosis.

## Materials and methods

### Animals

Six- to eight-week-old male BALB/c and C57BL/6 wild-type mice were used in these experiments. All the protocols were approved by the Animal Care and Use Committee of Qilu Hospital, and all animals were treated according to the ARVO Statement for the Use of Animals in Ophthalmic and Vision Research.

### Corneal injury and topical administration of acriflavine

Mice were anesthetized by intraperitoneal injection of Ketamine and Xylazine followed by one to two drops of topical 0.5% Proparacaine. Alkali burn induced corneal injury was generated in the right eye of each BALB/c mouse according to a previous report (Kethiri et al., 2019) with a minor modification. In brief, after the corneal and limbal epithelium was gently scraped by using a surgical scalpel No.15, topical application of 6 μl 0.1 M sodium hydroxide (NaOH) for 10 s was followed. The eye was rinsed immediately for 4 min with normal saline after the burn, and followed by topical application of 0.05% and 0.01% ACF (w/w, dissolved in PBS) twice daily, respectively. PBS was used as a control. A scoring system was used to evaluate the corneal neovascularization and corneal opacity. Neovascularization was scored on a scale of 0–4 which was assigned to the five areas of each cornea (central, superior, inferior, nasal, and temporal) based on the occupied area of the new blood vessels: 0, no new blood vessels; 1, less than 30% area; 2, more than 30% area but less than 70%; 3, more than 70% area less than 100%; and 4, 100% area. A total score of each cornea was calculated (range 0–20). The corneal opacity was also scored on a scale of 0–4 where 0 = completely clear; 1 = slightly hazy, iris and pupils easily visible; 2 = slightly opaque, iris and pupils still detectable; 3 = opaque, pupils hardly detectable, and 4 = completely opaque with no view of the pupils (Yoeruek et al., 2008). The healing percentage of the corneal epithelium was calculated using the NIH ImageJ software. The anterior chamber oxygen was measured using the DP-PSt7-2 oxygen sensor (PreSens, Regensburg, Germany).

A modified protocol of mechanical corneal injury was performed in the right eye of each C57BL/6 mouse as previously described (Mittal et al., 2016). In brief, the surface of the central cornea was demarcated with a 2-mm trephine followed by gentle scraping of the central epithelium with a surgical scalpel No. 15. Next, the basement membrane and anterior portion were removed using a hand-held Algerbrush II (Alger Equipment). Two days after the injury, 0.01% ACF was topical administrated twice daily. PBS was used as a control. Digital corneal images were taken by using a slit-lamp microscope and the degree of opacity was calculated using the NIH ImageJ software. Anterior segment images were taken using anterior segment-optical coherence tomography (OCT) (Bioptigen Inc., Durham, NC). Central corneal thickness was measured using the OCT built-in software.

### Immunofluorescent staining and histology

Cryosections of the whole eyeball were fixed in 4% paraformaldehyde for 20 min followed by incubating in 0.1% Triton X-100 and 2% BSA for 1 h at room temperature. The slides were incubated with anti-α-SMA (ab124964, Abcam), anti-fibronectin (15613-1-AP, ThermoFisher) and anti-CD45 (160302, Biolegend) antibodies at 4°C overnight, followed by immunostaining with Alexa Fluor 594 donkey anti-rabbit (A-21207, Invitrogen) or Alexa Fluor 488-donkey anti-rat (712-545-150, Jackson ImmunoResearch Europe Ltd.) secondary antibody for 1 h at room temperature. The slides were mounted with DAPI mounting medium (H-1200, Vector lab, Burlingame, CA) and photographed under a confocal laser scanning microscope (SP8, Leica, Wetzlar, Germany). For histological evaluation, corneal sections were stained with H&E and examined using bright-field microscopy (DMiL, Leica).

### *In vitro* corneal fibroblasts stimulation

Corneas were harvested from C57BL/6 wide-type mice and the Descemet’s-endothelium complex was stripped away with forceps. To loosen epithelial sheets, the remaining corneal stroma and epithelium were incubated in Dispase II (Roche) in Dulbecco’s modified Eagle’s medium (DMEM, Gibco) supplemented with 10% fetal bovine serum (FBS) and antibiotics (penicillin and streptomycin at 100 μg/ml) in 5% CO_2_ at 37°C. After removal of the epithelium, the stroma was cut into small segments and digested in DMEM containing 3.3 mg/ml collagenase type II (Sigma-Aldrich) at 37°C with shaking for 90 min. The isolated keratocytes were grown in DMEM supplemented with 10% FBS at 37°C in a 5% CO_2_ humidified atmosphere. One to three passage corneal fibroblasts were used for all experiments. The mouse corneal fibroblast was seeded at 1 × 10^5^ cells in 24-well plates and starved in DMEM containing 1% FBS overnight. Then the cells were cultured in medium alone or stimulated with 100 ng/ml murine recombinant TGF-β1 (R&D Systems) in the presence or absence of 0.0001% acriflavine (Sigma-Aldrich) for 24 h. Cells were then used for the evaluation of α-SMA, fibronectin, and the HIF-1 downstream genes of Bnip3 and Slc2a1 by immunocytochemistry and real-time PCR.

### Live/dead cells staining

The cell viability was evaluated using LIVE/DEAD™ Viability/Cytotoxicity Kit (L3224, Invitrogen) according to the product’s procedure. In brief, corneal fibroblasts cells were incubated for 30 min at room temperature with a mixture of 2 μM calcein acetoxymethyl ester (Calcein AM) and 4 μM ethidium homodimer-1 (EthD-1). The live cells (green fluorescence) and dead cells (red fluorescence) were photographed using fluorescence microscope (DMi8, Leica).

### RNA isolation and real-time PCR

Corneal tissues were harvested under dissecting microscope and placed in TRIzol solution (Invitrogen). Total RNA was isolated using the RNeasy Micro Kit (Qiagen) following the manufacturer’s protocol and reverse-transcribed to cDNA with QuantiTect Rev. Transcription Kit (Qiagen). Real-time PCR was then performed using Taqman Universal PCR Mastermix (ThermoFisher) and pre-formulated Taqman primers for murine glyceraldehyde-3-phosphate dehydrogenase (GAPDH), *β*-Actin (Mm02619580_g1), HIF-1α (Mm00468869_m1), α-SMA (Mm00725412_m1), fibronectin (Mm01256744_m1), Bnip3 (Mm01275600_g1), Slc2a1 (Mm00441473_m1), and VEGFA (Mm00437306_m1) (ThermoFisher). The results were analyzed by the comparative threshold cycle method and normalized to GAPDH or *β*-Actin as internal control.

### Flow cytometry

A single-cell suspension of corneal cells was prepared by digesting the individual cornea with Liberase TL (2.5 mg/ml) (Sigma-Aldrich) as previously reported (Gaddipati et al., 2015). Cells were stained with fluorochrome-conjugated monoclonal antibodies against CD45 (103116, Biolegend), HIF-1α (IC1935G, R&D system) and their isotype controls (400623, 400132, Biolegend). The stained cells were analyzed using the LSRII flow cytometer (BD Biosciences, San Jose, CA) and FlowJo software.

### Statistical analysis

The statistical analysis was conducted using GraphPad Prism software. Results are presented as means ± SEM. Comparisons between two groups were performed by an unpaired, two-tailed Student’s *t*-test; and comparison among three groups was performed by One-way ANOVA test. The statistically significant was set at *p* < 0.05.

## Results

### The HIF-1α level was elevated in the alkali burn injured corneal tissue

**FIGURE 1:**
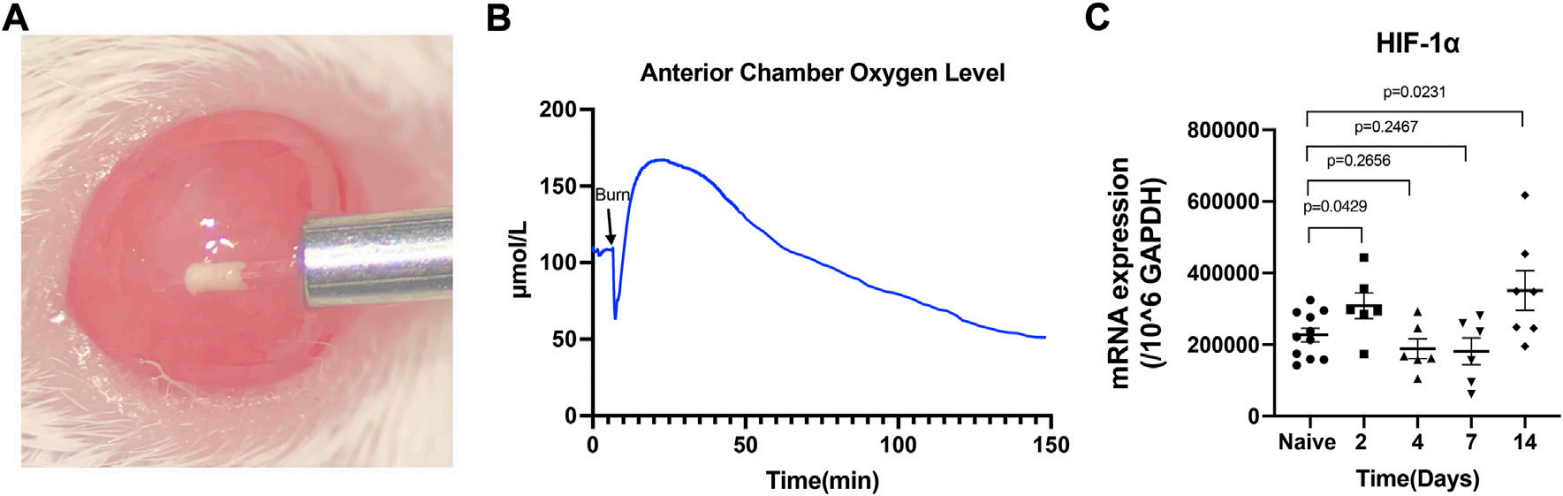
The alkali burn induced hypoxia in the mouse anterior chamber and elevated the HIF-1α mRNA level in the corneal tissue. Oxygen concentration in the anterior chamber **(B)** was recorded in real-time by inserting a micro-oxygen sensor **(A)**. The mRNA level of HIF-1α in naïve and day 2, 4, 7, 14 post-injured cornea was assayed by RT-PCR **(C)**. Data were presented as the mean ± SEM.

In the alkali burn induced mouse corneal injury models, the oxygen level in the anterior chamber was downregulated immediately after the burn and then increased to a higher level of around 160 μmol/L in 10 min due to the corneal epithelium disruption. After that, the oxygen level decreased smoothly to a low level of around 60 μmol/L in 2 h (Figures 1A,B). We detected the HIF-1α mRNA level during the 14 days of follow-up and found that the mRNA levels of HIF-1α were significantly increased at 2 days and 14 days (*p* = 0.0429 and *p* = 0.0231, respectively) compared to the naïve cornea after the burn (Figure 1C). The protein level of HIF-1α in the injured cornea was much higher than in native corneal tissue after the injury as demonstrated by flow cytometry analysis (Supplementary Figure S1). However, the significant difference in HIF-1α mRNA level compared to the naïve corneal tissue was not observed at both 4 and 7 days of follow-up.

### Acriflavine treatment delayed the corneal opacity and neovascularization after the burn

**FIGURE 2:**
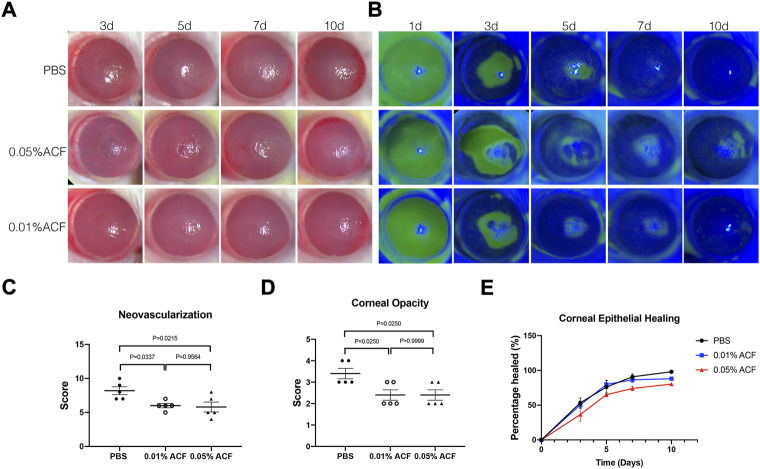
Topical administration of ACF delayed the corneal opacity and neovascularization after the alkali burn injury. **(A)** Slit-lamp photos of the alkali burn injury treated by 0.05% and 0.01% ACF. PBS treated group was as the control. A dose-dependent manner of delayed epithelium healing was observed in ACF treated group by fluorescein staining **(B,E)**. However, no significant difference was demonstrated among the three groups. On day 7 after the burn, both 0.05% and 0.01% ACF significantly reduced the neovascularization **(C)** and corneal opacity **(D)** compared to the control group. Data were presented as the mean ± SEM.

Besides limbal stem cell deficiency, neovascularization and corneal stromal fibrosis are both serious risk factors for blindness after alkali burn. Hence, we administered the drug ACF (0.01% and 0.05%, respectively) topically after the burn and determine how it worked in this model (Figure 2A). The scoring of corneal opacity and neovascularization were carried out in three groups at 7d post-injury. We found that both 0.01% and 0.05% ACF inhibited the corneal neovascularization (*p* = 0.3337 and *p* = 0.0215, respectively) and opacity (*p* = 0.0250 and *p* = 0.0250, respectively) compared to PBS treated group (Figures 2C,D). However, there were no significant differences in these three groups 10 days after alkali burn (data not shown). We also observed that ACF delayed epithelial healing in a dose-dependent manner by fluorescein staining (Figures 2B,E). Although the ACF drugs inhibited the hemangiogenesis and corneal opacity at the early stage after the alkali burn, no significant difference was observed in a long-term follow-up. The reason might be the limbal stem cells deficiency and corneal conjunctivalization in the chronic-stage post-injury that the independent inhibition of HIF could not overcome these pathological processes.

### Topical administration of acriflavine restored the corneal transparency in mechanical ocular injury

**FIGURE 3:**
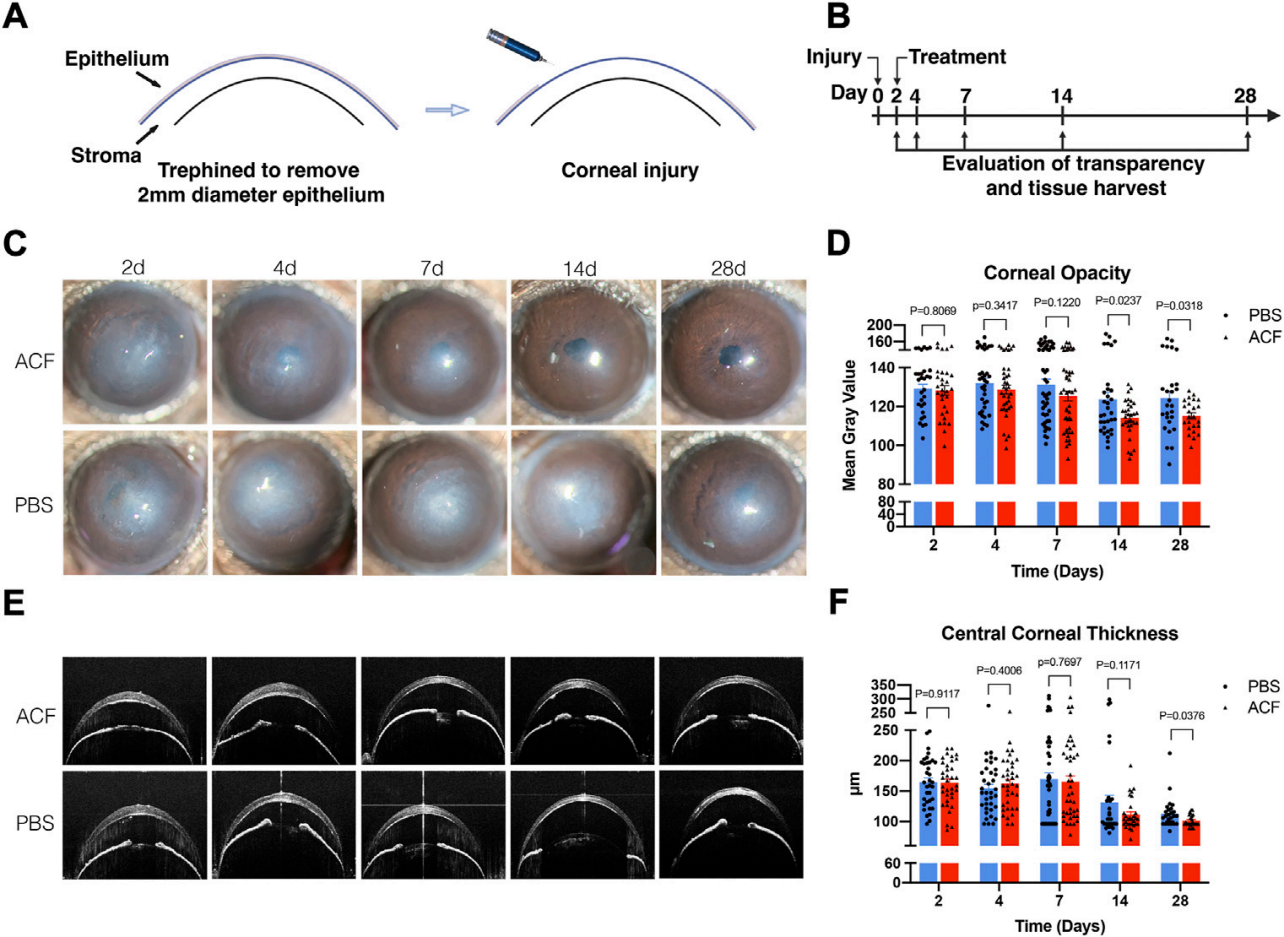
Topical administration of ACF restored the corneal transparency in mechanical ocular injury. The corneal injury was induced by the mechanical removal of the corneal epithelium and anterior stroma **(A)**. Two days after the injury, 0.01% ACF was topically administrated twice daily and followed for 28 days **(B)**. Slit-lamp photos demonstrated that ACF significantly reduced the corneal scarring and restored the corneal transparency at day 14 and 28 post-injury compared to PBS treated group **(C)**. The corneal opacity was analyzed by ImageJ **(D)**. The central corneal thickness was examined by OCT **(E)**, and showed a reduction 28 days after the injury **(E,F)**. Data were presented as the mean ± SEM.

To determine whether topical ACF treatment has the potential to restore the corneal transparency following injury, we utilized a well-characterized sterile injury model of mouse cornea (Mittal et al., 2016) with a minor modification, as details were described in the Materials and methods. The injury was induced by the mechanical removal of the corneal epithelium and anterior stroma (Figure 3A); 2 days after the injury, topical 0.01% ACF was administrated twice daily (Figure 3B). Topical administration of PBS was used as the control group. Slit-lamp biomicroscopy was used to monitor the extent of corneal opacity and analyzed by ImageJ (NIH). The central corneal thickness was evaluated by OCT. On day 2 post-injury, both groups showed a significant development of corneal opacity. However, the corneas of mice administrated with topical 0.01% ACF showed a significant reduction in corneal opacity at days 14 and 28 post-injury compared to PBS treated group (*p* = 0.0237 at days 14; *p* = 0.0318 at days 28; Figures 3C,D). Moreover, the central corneal thickness was also significantly reduced at 28 days (101.3 ± 9.4 μm in ACF treated group and 112.7 ± 24.5 μm in the control group, *p* = 0.0376; Figures 3E,F).

### Acriflavine treatment inhibited the generation of fibrosis in the injured corneal stroma

**FIGURE 4:**
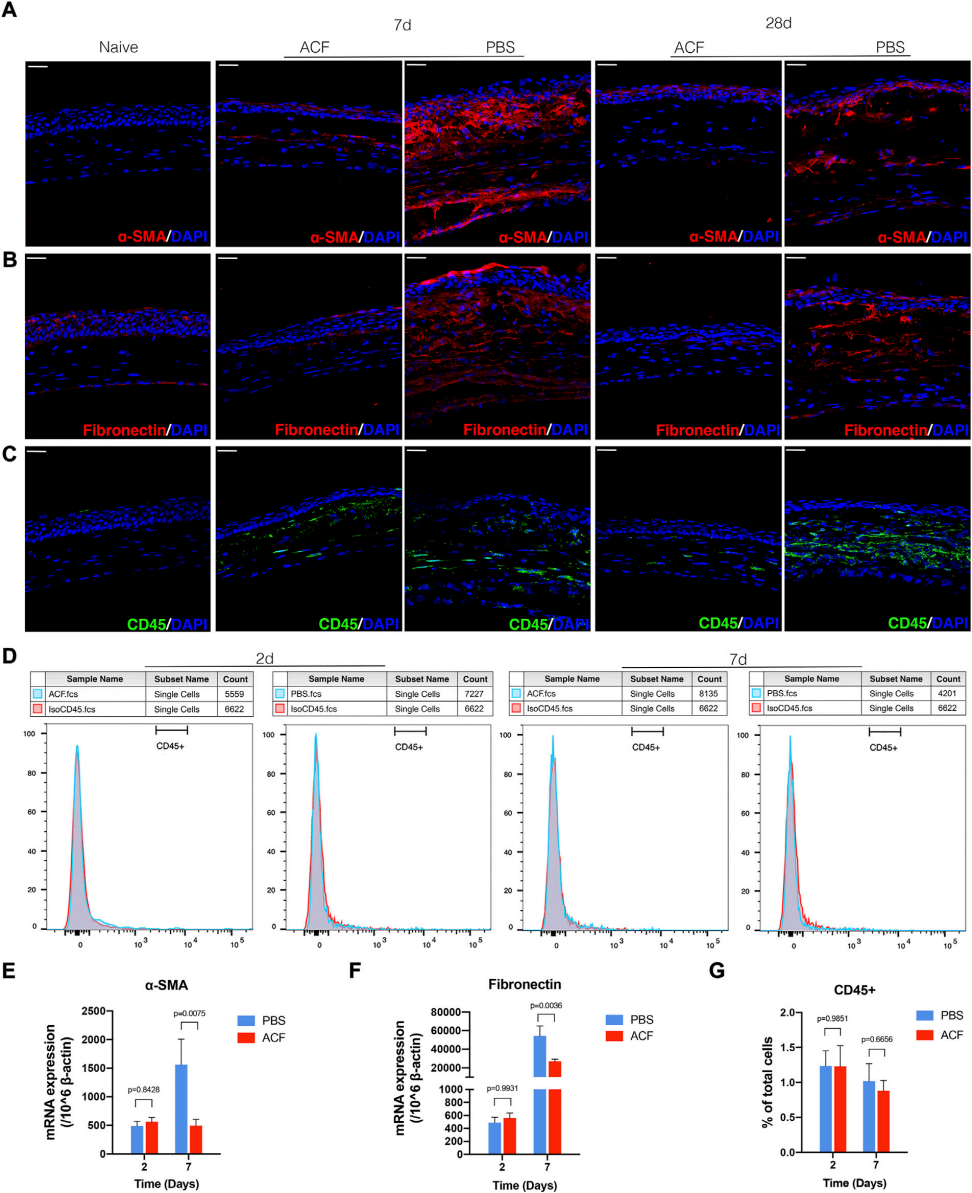
ACF inhibited the generation of fibrosis in the injured corneal stroma. Tissues were harvested at day 7 and 28 for immunohistochemistry evaluation. The immunofluorescent staining of α-SMA (red) and fibronectin (red) in corneal stroma were much higher in PBS treated control group **(A,B)**. The mRNA level of α-SMA and fibronectin at day 7 post-injury was consistent with the immunofluorescent staining result **(E,F)**. The infiltration of CD45^+^ leucocytes in the corneal tissues was evaluated by immunofluorescent staining [**(C)**, green] and flow cytometry **(D,G)**, respectively. No significant difference was observed between the two groups at day 2 and 7 after the injury. Data were presented as the mean ± SEM. Scale bar: 10 μm.

To confirm the efficacy of ACF in reducing corneal opacity formation and preserving transparency, the corneal tissues were harvested at days 2, 7, 14, and 28 post-injury to determine the expression of α-SMA and fibronectin at cellular and molecular levels in the corneal stroma. The confocal micrographs of immunofluorescent staining corneas showed a significant reduction in the expression of α-SMA and fibronectin in the ACF-treated corneas at 7, 14, 28 days post-injury compared with control corneas (Figures 4A,B; Supplementary Figures S2, S3B,C). These findings were also confirmed by real-time PCR in two groups 7 days after injury (*p* = 0.0075 and *p* = 0.0036, respectively; Figures 4E,F). H.E. staining of corneal cross-sections revealed the increased stratification of the epithelial cell layer in the ACF-treated cornea (Supplementary Figure S4). We also examined the infiltration of leucocytes in the injured corneal stroma at two- and seven-days post-injury and did not find significant difference between the two groups (Figures 4C,D,G).

### Inhibition of hypoxia-inducible factors reduced the expression of α-SMA and fibronectin in the corneal fibroblasts stimulated by TGF-β1

**FIGURE 5:**
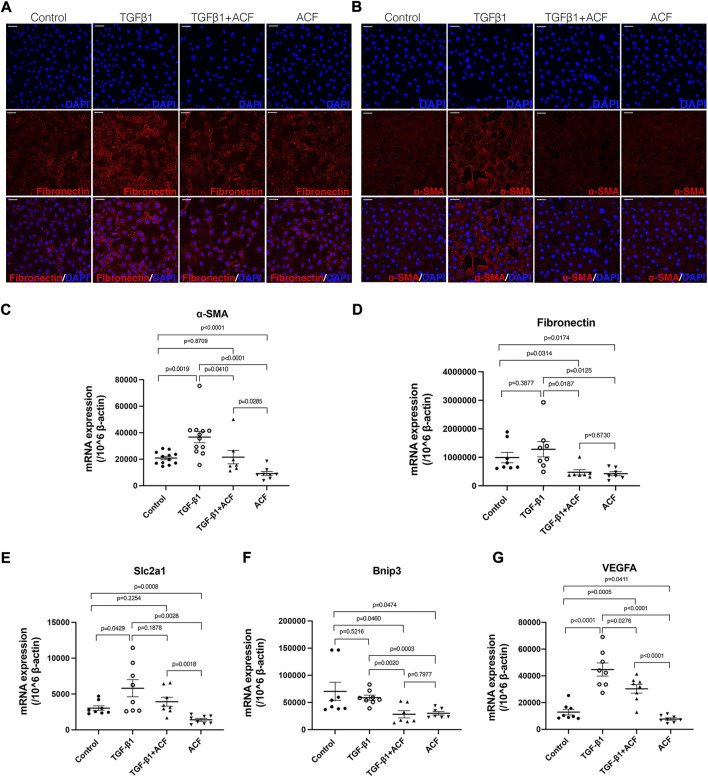
ACF inhibited the expression of α-SMA and fibronectin in the corneal fibroblasts. The corneal fibroblasts were cultured in medium alone or stimulated with 100 ng/ml murine recombinant TGF-β1 in the presence or absence of 0.0001% ACF for 24 h. **(A,B)** are the immunofluorescent staining of fibronectin (red) and α-SMA (red), respectively. The mRNA level of α-SMA was significantly reduced in the presence of ACF with or without TGF-β1 stimulation **(C)**. The mRNA level of fibronectin was not affected by TGF-β1 but reduced by the presence of ACF **(D)**. The mRNA level of HIF-1α downstream gene Slc2a1 was elevated by TGF-β1 stimulating **(E)**. ACF inhibited the Slc2a1 mRNA level without the presence of TGF-β1 **(E)**. The other HIF-1α downstream genes bnip3 **(F)** and VEGFA **(G)** were significantly downregulated by the presence of ACF. Data were presented as the mean ± SEM. Scale bar: 10 μm.

To determine whether ACF can directly inhibit the expression of α-SMA and fibronectin in the corneal fibroblasts, we stimulated the mouse corneal fibroblast with TGF-β1 in the absence or presence of ACF for 24 h. The unstimulated cultures served as a control group. Before this study, we analyzed the cell viability after the ACF treatment by using a Live/Dead Viability/Cytotoxicity Kit, and found that ACF did not affect the cell viability (Supplementary Figure S5). ACF suppressed the TGF-β1-induced α-SMA expression in corneal fibroblasts by the examination of the immunofluorescent staining and real-time PCR (Figures 5B,C). TGF-β1 stimulation could not promote the mRNA level of fibronectin in the corneal fibroblasts (*p* = 0.3877, Figure 5D). However, ACF treatment significantly reduced the baseline level of fibronectin as well as α-SMA in corneal fibroblasts (Figures 5A,C,D), suggesting that ACF could be effective in reversing pre-formed myofibroblasts into α-SMA negative fibroblasts and reducing the fibrosis. In addition, we found that the mRNA level of HIF-1α downstream gene Slc2a1, responsible for the production of glucose transporter protein-1 (GLUT-1), and the VEGFA, were both elevated by TGF-β1 stimulation. ACF down-regulated the Slc2a1 and VEGFA mRNA levels without the presence of TGF-β1 (Figures 5E,G). TGF-β1 had no effect on another HIF-1α downstream gene bnip3. However, it was significantly downregulated by the presence of ACF (Figure 5F).

## Discussion

The fibrosis of the cornea can occur for many reasons including chemical burn, infections, trauma, inflammation, surgeries, or other corneal diseases, leading to corneal blindness (Wilson et al., 2012). TGF-β signaling pathway is the most well-known and robust inducer of fibrosis in the cornea (Bonnans et al., 2014). In the healthy cornea, the keratocytes in the stroma are quiescent and responsible for maintaining the balance of the stromal extracellular matrix (ECM) which is vital to corneal transparency (Pei et al., 2004). Upon injury, the keratocytes are driven by the TGF-β that enters the stroma mainly from the corneal epithelium and tears, and transdifferentiate into fibroblasts or myofibroblasts, thereby synthesizing a disorganized opaque matrix (such as fibronectin, type III collagen, tenascin C etc.) (de Oliveira et al., 2021). The α-SMA expressing myofibroblast is mainly responsible for corneal fibrosis, and sometimes is differentiated from other cells (e.g., bone marrow-derived fibrocytes) in the stroma (Kempuraj and Mohan, 2022; Wilson et al., 2022). Corneal hypoxia caused by contact lens wear or inflammation, usually results in corneal neovascularization (CNV), which often severely compromises vision by decreasing the transparency of corneal stroma (Safvati et al., 2009). The CNV-associated pericytes are α-SMA positive and have been found to have the capacity to transdifferentiate into myofibroblasts (Kostallari and Shah, 2019; Wilson et al., 2022). Therefore, under hypoxia conditions or serious corneal damage, multiple studies focused on the inhibition of CNV to prevent corneal opacity by blocking vascular endothelial growth factor (VEGF) receptors, attenuating inflammation or targeting the VEGF upstream regulator of HIF-1α (Peral et al., 2022).

HIF, a basic loop-helix-loop protein that forms a heterodimeric complex, is a crucial mediator of the hypoxic response, which transactivates a wide range of downstream genes including those promoting angiogenesis, anaerobic metabolism, resistance to apoptosis and potentially profibrotic (McNutt, 2013; Palazon et al., 2014). These genes include VEGF, erythropoietin (EPO), GLUT-1, Bnip3, connective tissue growth factor (CTGF), transforming growth factor-β1, platelet-derived growth factor (PDGF), etc. (Darby and Hewitson, 2016). Besides hypoxia, bacteria (Peyssonnaux et al., 2005), inflammatory cytokines (e.g., TNF-α, NF-κB, IL-6, IL-4) (Albina et al., 2001; Rius et al., 2008; Takeda et al., 2010; Dang et al., 2011) and reactive oxygen species (ROS) (Shatrov et al., 2003) were found to be responsible for HIF-1α or HIF-2α stabilization, which indicated that HIFs might play wide range roles in physiological function (Palazon et al., 2014). Noteworthily, normoxic TGF-β stimulation increased HIF-1α expression in human kidney epithelial cells (Basu et al., 2011). The increased production of matrix components such as fibronectin and collagen following the stabilization of HIF-1 in dermal fibroblasts has also been demonstrated (Darby and Hewitson, 2016). In most tissues, progressive fibrosis is observed if hypoxia persists (Ballermann and Obeidat, 2014). The fibrosis inhibition has also been noted in kidney and lung tissues by reducing or inhibiting the nuclear localization of HIF-1α or HIF-2α (Tanaka, 2016; Fu et al., 2021). As demonstrated previously, the blocking or reduction of HIF-1/2 signaling pathways in both renal and dermal cells has a negative effect on collagen synthesis and on the TGF- β stimulation of collagen synthesis (Darby and Hewitson, 2016). Hence the HIF pathways might be a potential target for inhibiting fibrosis.

The nuclear localization of HIF-1α and HIF-2α protein was observed in Herpes stromal keratitis (HSK)-developing corneas (Rao and Suvas, 2019). In this model, the stabilization of HIF-2α protein was detected in corneal epithelial cells, whereas HIF-1α protein stabilization was observed in infiltrating cells. Interestingly, in the alkali-burn-induced mouse limbal stem cell deficiency (LSCD) mouse model, we also observed that the burn induced the hypoxia in anterior chamber and significantly increased the mRNA levels of HIF-1α in the corneas at 2 and 14 days follow-up. As described before, HIF-1α was highly expressed in the inflammatory cells (Rao and Suvas, 2019). The elevation of HIF-1α 2 days after the burn might be due to the infiltration of leukocytes. After 14 days, the CNV fully covered the cornea and induced a second peak expression of HIF-1α. In order to determine how the HIFs worked in this model, we topically administrated acriflavine (ACF), a drug inhibiting HIF DNA-binding and transcriptional activity (Lee et al., 2009), and found that ACF delayed the CNV formation and corneal opacity during the follow-up. However, due to the combination of limbal stem cells dysfunction (LSCD) after the alkali burn, intervention on HIF signaling alone might not enough for the inhibition of CNV and improvement of corneal transparency in a long-term follow-up.

In order to reduce the effect of LSCD on the results and detect whether HIF signaling played a role in corneal fibrosis formation, we utilized a mechanical corneal injury mouse model according to a previous report (Mittal et al., 2016). Since ACF delayed the epithelium closure in a dose-dependent manner from LSCD models, we treated the mechanical corneal injury with a lower dose of ACF after the epithelium fully closed 2 days after the injury. Noteworthily, the topical administration of ACF significantly improved the corneal transparency beginning at days 14 post-injury. The inhibition of corneal fibrosis was confirmed by immunohistochemistry and real-time PCR that the expression of α-SMA and fibronectin were both reduced in the ACF-treated corneal stroma. To further confirm the effect of the ACF and HIF pathways on corneal fibrosis, we cultured the corneal fibroblasts *in vitro* followed by stimulating with TGF-β1 with or without ACF. Similar to the *in vivo* results, ACF reduced the expression of α-SMA and fibronectin in both TGF-β1 stimulated or unstimulated fibroblasts. The HIF-1α downstream genes of Slc2a1, Bnip3 and VEGFA were also downregulated, and indirectly confirmed that ACF inhibited corneal fibrosis by working on HIF pathways. These data were consistent with previous reports in other tissues (McNutt, 2013; Palazon et al., 2014; Tanaka, 2016; Fu et al., 2021). However, Rao and Suvas (2019) demonstrated a different result that the systemic administration of ACF increased the influx of neutrophils in progressing herpes stroma keratitis lesions, resulting in increased corneal opacity. We also detected the infiltration of leukocytes in the mechanical injured corneal stroma and did not find any significant differences in ACF treated and PBS treated control group. Due to the different models and administration approaches between these two studies, HIF signals might play different roles in different etiologies of corneal stroma damage.

However, this study has drawbacks that need further investigation. As previously mentioned, the enhancement of hypoxia in the myofibroblast transdifferentiation has been found in many tissues, such as sclera, skin, etc. We detected that hypoxia and the elevated mRNA level of HIF-1α existed in the alkali burn injured corneal tissue, but we did not know exactly whether and how hypoxia directly affected the function and characteristics of keratocytes or fibroblasts in the injured corneal tissue or the formation of the corneal fibrosis. The *in vivo* or *in vitro* phenotypes and functions of keratocytes under hypoxia conditions are needed for further observation. In addition, although we observed that the inhibition of HIFs by using ACF improved the corneal transparency post-injury and reduced the transdifferentiation of myofibroblast, we did not know whether and how HIFs enhanced the fibrosis formation in the cornea stroma. For example, we should know if the elevation of HIFs in corneal tissue or keratocytes promoted corneal fibrosis or myofibroblast transdifferentiation *in vivo* and *in vitro*. Moreover, additional studies are needed to determine the precise mechanisms of the relationship between HIF and TGF-β1 pathways as well as the inhibition of fibronectin production by HIFs through which pathways.

In summary, we demonstrated that the inhibition of HIF signals reduced the corneal fibrosis in the mechanical damaged corneal stroma and improved corneal transparency. The ACF could be effective in reversing pre-formed myofibroblasts into α-SMA negative fibroblasts and reducing the corneal fibrosis. This mechanism involved the TGF-β1 pathways. Moreover, based on our study, inhibition of HIF signals also reduced the fibronectin expression in corneal fibroblast and deposition in corneal stroma, which was not TGF-β1 dependent. This is the first study to implicate that HIFs might be a new treatment target for controlling corneal fibrosis in mechanical corneal injuries.

## Data Availability

The original contributions presented in the study are included in the article/Supplementary Material, further inquiries can be directed to the corresponding author.
