# Corrigendum: Therapeutic potential of topical administration of acriflavine against hypoxia-inducible factors for corneal fibrosis

**DOI:** 10.3389/fphar.2023.1161682

**Published:** 2023-02-21

**Authors:** Shuyan Zhu, Huimin Shan, Jianqiao Li, Lijie Pan, Shudan Wang, Jing Zhu, Hui Guo, Fenghua Mi, Xinyi Wu, Jia Yin, Kunpeng Pang

**Affiliations:** ^1^ Xi’an People’s Hospital (Xi’an Fourth Hospital), Shanxi Eye Hospital, Xi’an, China; ^2^ Department of Ophthalmology, Qilu Hospital of Shandong University, Jinan, Shandong, China; ^3^ Department of Ophthalmology, Harvard Medical School, Schepens Eye Research Institute of Massachusetts Eye and Ear, Boston, MA, United States

**Keywords:** HIF, corneal fibrosis, corneal hypoxia, TGF-β1, extracellular matrix

In the published article, there was an error in the **Author** list, and author “Jia Yin” was erroneously excluded. The corrected **Author** list appears below.

“Shuyan Zhu^1^, Huimin Shan^2^, Jianqiao Li^2^, Lijie Pan^2^, Shudan Wang^3^, Jing Zhu^2^, Hui Guo^2^, Fenghua Mi^2^, Xinyi Wu^2^, Jia Yin^3^*, Kunpeng Pang^2*^.”

In the published article, Jia Yin’s information was not included in the correspondence information. The corrected **Correspondence** information is “Jia Yin, jia_yin@meei.harvard.edu; Kunpeng Pang, k.pang@sdu.edu.cn.”

In the published article, the **Author Contributions** statement did not mention Jia Yin’s contributions. The correct statement is “JY and KP contributed to conception and design of the study. KP supervised the study. SZ, HS, SW, and KP conducted experiments and acquired data; HS and KP wrote the manuscript; LP analyzed data; FM assisted in conducting the experiments; JZ and HG assisted in analyzing data; JL and XW provided the reagents; KP designed research studies and revised the manuscript.”

In the published article, the **Acknowledgements** statement was displayed as “We thank Dr. Jia Yin as an advisor for the hypoxia study.” The correct statement is “None.”

The authors apologize for this error and state that this does not change the scientific conclusions of the article in any way. The original article has been updated.

