# Supplementary material for: Therapeutic potential of topical administration of acriflavine against hypoxia-inducible factors for corneal fibrosis
Source: Front Pharmacol. 2022 Oct 21;13:996635. doi: 10.3389/fphar.2022.996635 (PMC9634531; doi:10.3389/fphar.2022.996635)
Supplement: Supplementary file 1 [file Table1.DOCX]

**Supplemental figures**

**
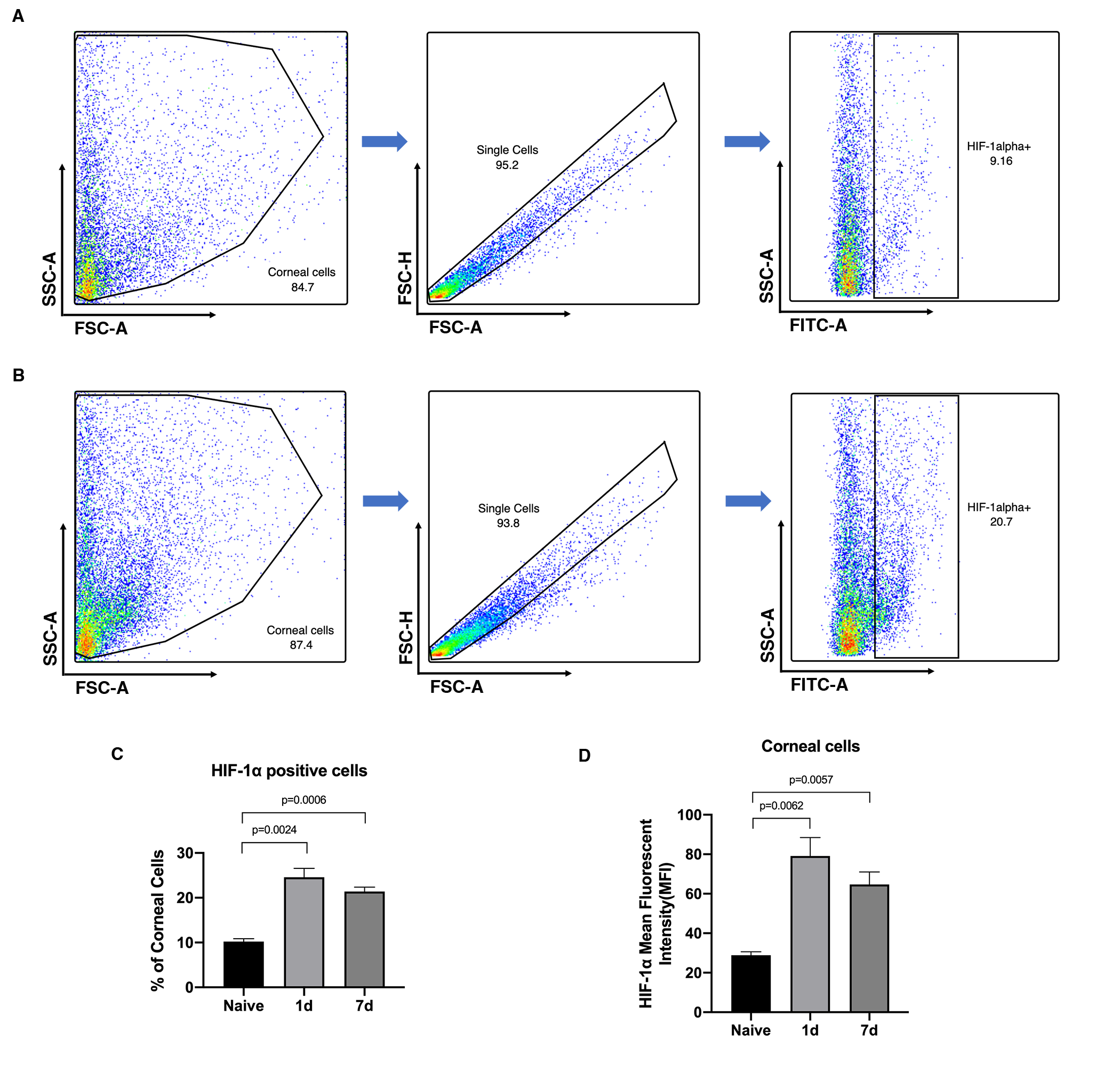
**

**Figure S1. The flow cytometry analysis of HIF-1α in the alkali burn injured cornea.** A: Naïve cornea; B: One day after the burn; C: The percentage of HIF-1α positive cells; D: HIF-1α mean fluorescence intensity (MFI) of the corneal cells.


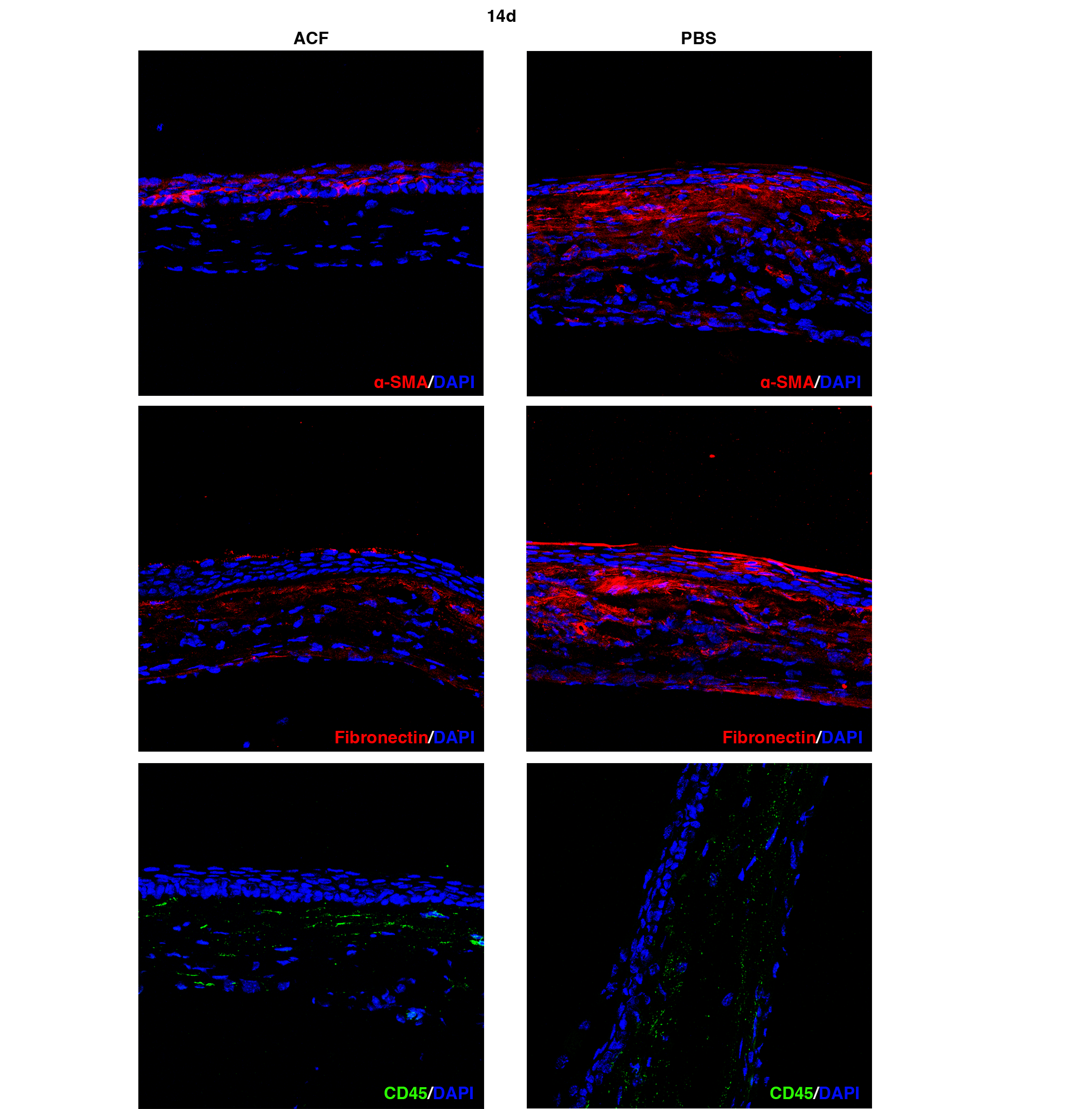


**Figure S2. The immunofluorescent staining of the cornea 14 days after the injury.** The left column is the ACF-treated group, and the right column is the PBS-treated control group.

**
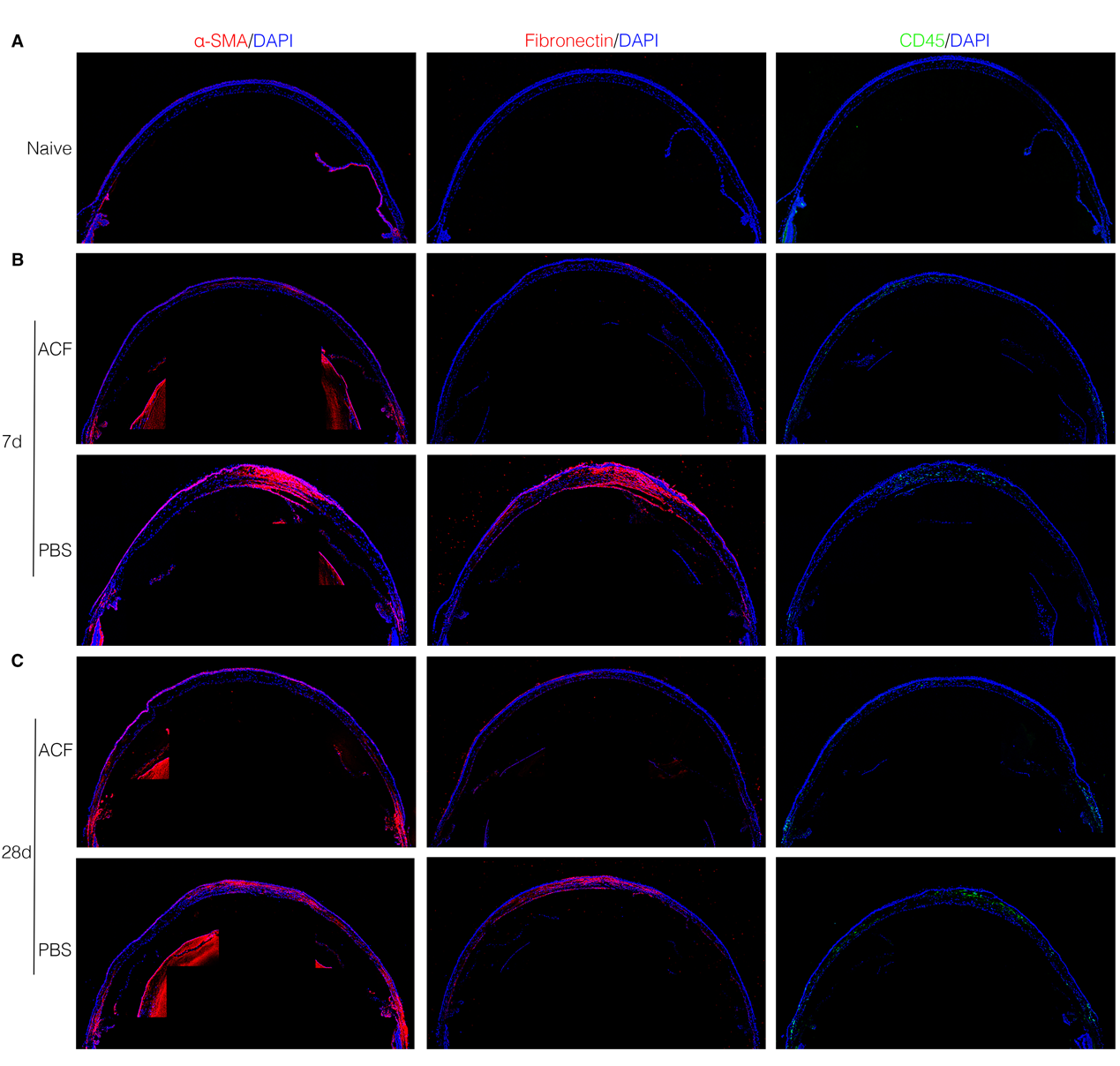
**

**Figure S3. The immunofluorescent staining of the anterior segment of the eyeball**. Alpha-SMA (red) and fibronectin (red) were highly expressed in PBS treated corneal stroma compared to the ACF treated group at both day 7 and 28 post-injury, and they were negative in naïve corneal stroma. The infiltration of CD45+ leukocytes (green) was higher in both mechanical corneal stoma groups than it was in naïve cornea.

**
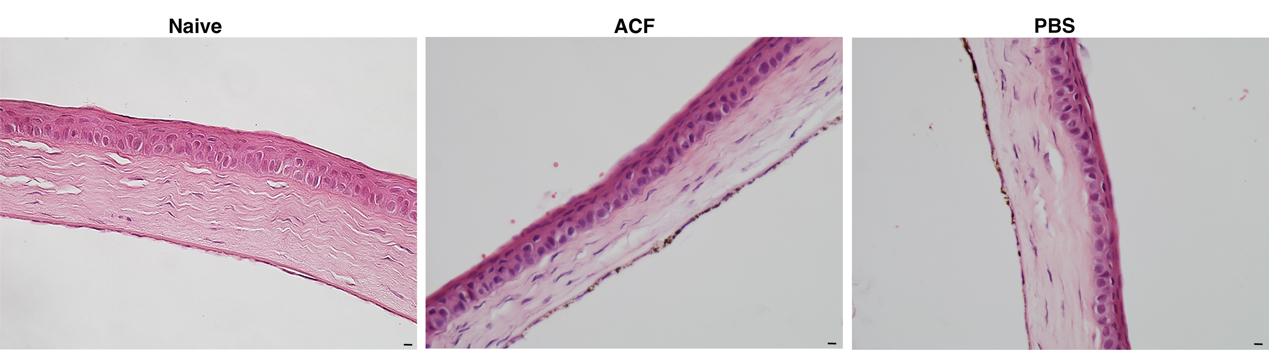
**

**Figure S4**. **H.E. staining of the mechanically injured cornea.** Twenty-eight days after the injury, eyeballs were collected for frozen section and H.E. staining. The increased stratification of the epithelial cell layer in the ACF-treated cornea was observed compared to the PBS-treated control group.

**
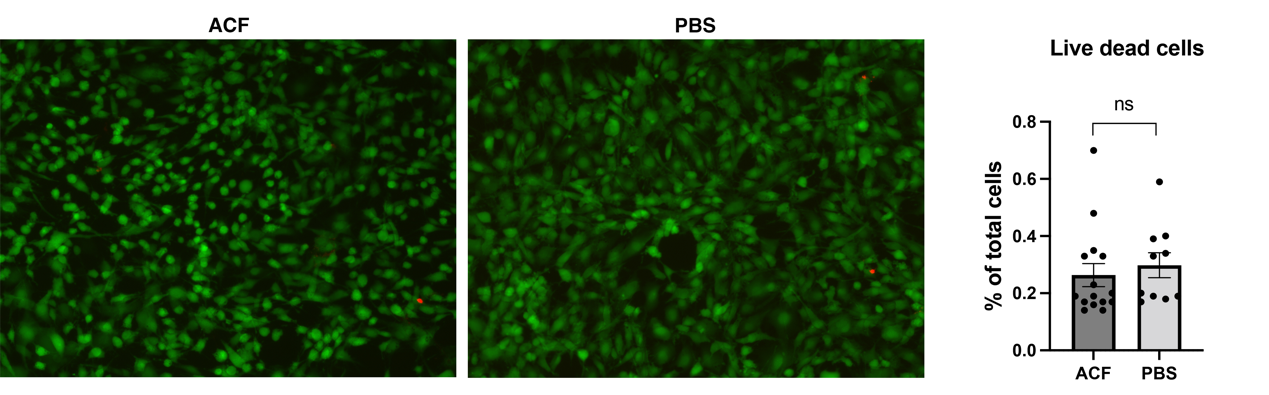
**

**Figure S5. Live dead cells staining of the corneal fibroblasts.** The live cells were stained green, and the dead cells were stained red. No significant difference was found between the ACF and PBS treated groups.
